# Satb2 regulates the development of dopaminergic neurons in the arcuate nucleus by Dlx1

**DOI:** 10.1038/s41419-021-04175-9

**Published:** 2021-09-25

**Authors:** Qiong Zhang, Lei Zhang, Ying Huang, Pengcheng Ma, Bingyu Mao, Yu-Qiang Ding, Ning-Ning Song

**Affiliations:** 1 Department of Laboratory Animal Science, Fudan University, Shanghai, China; 2 Key Laboratory of Arrhythmias, Ministry of Education of China, East Hospital, and Department of Anatomy and Neurobiology, Tongji University School of Medicine, Shanghai, China; 3 State Key Laboratory of Medical Neurobiology and MOE Frontiers Center for Brain Science, Institutes of Brain Science, Fudan University, Shanghai, China; 4 Department of Anatomy, Histology and Embryology, School of Basic Medical Sciences, Fudan University, Shanghai, China; 5 State Key Laboratory of Genetic Resources and Evolution, Kunming Institute of Zoology, Chinese Academy of Sciences, Kunming, China; 6 Center for Excellence in Animal Evolution and Genetics, Chinese Academy of Sciences, Kunming, China

**Keywords:** Neuronal development, Brain

## Abstract

Dopaminergic (DA) neurons in the arcuate nucleus (ARC) of the hypothalamus play essential roles in the secretion of prolactin and the regulation of energy homeostasis. However, the gene regulatory network responsible for the development of the DA neurons remains poorly understood. Here we report that the transcription factor special AT-rich binding protein 2 (Satb2) is required for the development of ARC DA neurons. Satb2 is expressed in a large proportion of DA neurons without colocalization with proopiomelanocortin (POMC), orexigenic agouti-related peptide (AgRP), neuropeptide-Y (NPY), somatostatin (Sst), growth hormone-releasing hormone (GHRH), or galanin in the ARC. Nestin-Cre;Satb2^flox/flox^ (Satb2 CKO) mice show a reduced number of ARC DA neurons with unchanged numbers of the other types of ARC neurons, and exhibit an increase of serum prolactin level and an elevated metabolic rate. The reduction of ARC DA neurons in the CKO mice is observed at an embryonic stage and Dlx1 is identified as a potential downstream gene of Satb2 in regulating the development of ARC DA neurons. Together, our study demonstrates that Satb2 plays a critical role in the gene regulatory network directing the development of DA neurons in ARC.

## Introduction

Hypothalamus, in particular the arcuate nucleus (ARC), is critical for the regulation of feeding and metabolism [1]. Two major types of neurons are identified in the ARC: neurons expressing the anorexigenic α-melanocyte-stimulating hormone derived from POMC, and those expressing AgRP or NPY [2]. Generally, POMC neurons promote activity, energy expenditure and suppress food intake, while AgRP/NPY neurons suppress energy use and promote food intake. In addition, the ARC also contains other types of neurons, such as those expressing somatostatin (Sst), growth hormone-releasing hormone (GHRH), galanin, and dopamine, and many non-POMC neurons contain and release inhibitory neurotransmitter γ-aminobutyric acid (GABA) [3].

Dopamine is an important neurotransmitter in the central nervous system. ARC dopaminergic (DA) neurons in the hypothalamus are divided into the dorsomedial (DM) and ventrolateral (VL) parts based on their location, soma size, and neurochemical characteristics [4, 5]. The ARC DA neurons project to and release dopamine in the median eminence, and released dopamine is transported to the anterior pituitary gland via the portal system. One of the well-known physiological roles of ARC DA neurons is to suppress the secretion of prolactin from prolactin-producing cells (lactotrophs) in the anterior pituitary gland through activating dopamine receptor 2 (D2) receptors. Recently, ARC DA neurons are also implicated in energy homeostasis [6]. Previous studies have revealed that Mash1 [7], Nkx2.1 [8], Islet1 [9], and Dlx1/2 [10] are required for the morphogenesis of ARC or the production of ARC neuroendocrine cells including DA neurons. However, the molecular mechanisms that selectively regulate the development of ARC DA neurons remain elusive.

During cortical development, the transcription factor Satb2 (special AT-rich binding protein 2) determines the cell fate of callosal projection neurons [11, 12], controls the regionalization of the retrosplenial cortex [13], and soma spacing and dendritic self-avoidance of pyramidal neurons in the neocortex [14]. Our previous study has shown that Satb2 is expressed in a high proportion of ARC DA neurons [15], however, the function of Satb2 in the development of ARC DA neurons has not been reported.

In this study, Nestin-Cre mouse line was used to delete Satb2 in the mouse brain. Satb2 CKO (Nestin-Cre;Satb2^flox/flox^) mice exhibited reduced body weight and elevated metabolic rate. The development of ARC DA neurons was impaired, with normal appearance of other neuron types expressing POMC, AgRP/NPY, GAD67, Sst, GHRH, and Galanin, indicating a specific role of Satb2 in the development of ARC DA neurons. We also showed that serum prolactin level was increased in Satb2 CKO mice. Finally, we revealed that Dlx1 is likely to be a downstream gene of Satb2 in regulating the development of ARC DA neurons.

## Results

### Lower body mass and higher metabolic rate in Satb2 CKO mice

**Fig. 1:**
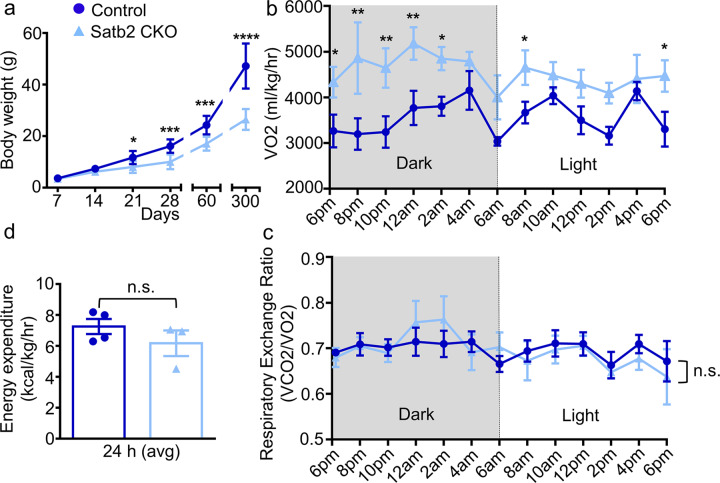
Lower body mass and higher metabolic rate in Satb2 CKO mice. **a** The body weight of Satb2 CKO mice is sharply reduced when compared with control mice during postnatal development, at adulthood, and at the aged stage. At P7-P60, *n* = 8 for control and *n* = 6 for Satb2 CKO mice; at P300, *n* = 11 for control and *n* = 7 for Satb2 CKO mice. Error bars represent S.D. **b** Oxygen consumption (VO2) was measured by indirect calorimetry in control and Satb2 CKO mice. Calorimetry was performed for 24 h spanning both the dark (12 h) and light (12 h) cycles. **c** RER was measured based on the oxygen and carbon dioxide data obtained by indirect calorimetry and showed no significant difference between control and Satb2 CKO mice. **d** The energy expenditure data averaged for 24 h are comparable between control and Satb2 CKO mice. For **b**–**d** *n* = 4 for control and *n* = 3 for Satb2 CKO mice and error bars represent S.E.M. Statistical differences were determined by Student’s *t*-test, *p < 0.05, **p < 0.01, ***p < 0.001, ****p < 0.0001; n.s. not statistically significant.

Previous studies have shown that Satb2 is critical for cortical development [11–14, 16–18]. However, the role of Satb2 in other brain regions is largely unknown. To this end, Satb2 floxed mice [19] were crossed with Nestin-Cre mice [20] to knock out this gene in the central nervous system. Littermates with other genotypes (i.e., Satb2^flox/+^ and Satb2^flox/flox^) had no alterations examined below and were used as controls. Satb2 CKO mice were viable and could survive into the adult stage, but showed reduced body weight during postnatal development and at adult and aged stages compared to their littermate controls (Fig. 1a). To investigate if the lower body weight was due to changes in basal metabolic rate, we placed the Satb2 CKO and control mice in the metabolic chambers and measured the oxygen consumption (VO_2_) and carbon dioxide expiration (VCO_2_) for 24 h by indirect calorimetry. Consistent with the reduction in body weight, Satb2 CKO mice exhibited a significant increase in VO_2_ during the total 24 h period including the dark and light cycles (Fig. 1b), indicating a higher basal metabolic rate in these mice. However, we did not detect a change in the respiratory exchange rate (RER, VCO_2_/VO_2_) (Fig. 1c) or energy expenditure (Fig. 1d). Taken together, our data revealed a higher metabolic rate and growth retardation in Satb2 CKO mice.

### DA neurons are selectively reduced in ARC of adult Satb2 CKO mice

**Fig. 2:**
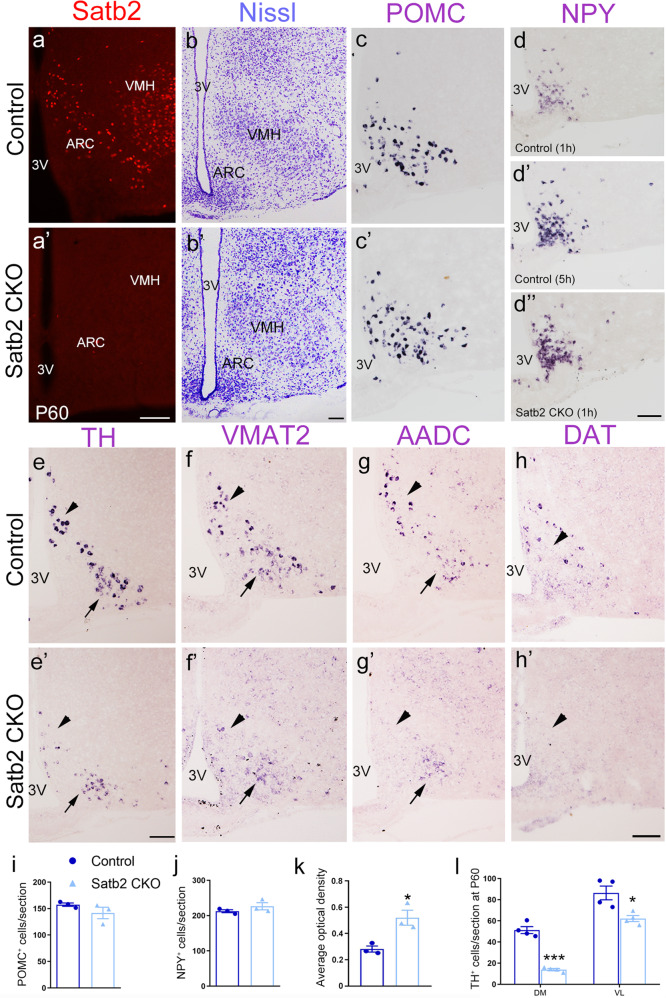
TH, not POMC or NPY, is selectively reduced in ARC of adult Satb2 CKO mice. **a**, **a’** Immunostaining shows the presence of Satb2 in the ARC and VMH of control but not Satb2 CKO mice at P60. **b**, **b’** Nissl-stained cellular architecture is not changed in the ARC of Satb2 CKO mice relative to controls. **c**, **c’** POMC^+^ neurons in the ARC are comparable between control and Satb2 CKO mice. **d**–**d”** The number of NPY^+^ neurons is comparable between control and Satb2 CKO mice, but the intensity of expression is higher in Satb2 CKO mice than that in control when compared at the same duration of signal development (**d**, **d”**). **e**, **e’** TH is expressed in both DM (triangle) and VL (arrow) of control mice, whereas its expression is dramatically reduced in the ARC particularly in the DM (triangle) of Satb2 CKO mice. **f**–**g’** The expression of DA neuron-associated genes in adult control and Satb2 CKO mice. VMAT2 and AADC are expressed in both DM (triangle) and VL (arrow) of control mice (**f**, **g**), whereas their expressions are dramatically reduced in the ARC particularly in the DM of Satb2 CKO mice (**f’**, **g’**). **h**, **h’** DAT is primarily expressed in the DM (triangle) of control mice (**h**), and its expression is hardly detected in Satb2 CKO mice (**h’**). **i**, **j** The quantification of POMC^+^ (**i**) and NPY^+^ (**j**) cells in the ARC shows no significant difference in cell number between the two groups. *n* = 3 for each group. **k** The quantification of intensity of NPY expression shows it is higher in Satb2 CKO mice than that in control mice. *n* = 3 for each group. **l** The quantification of TH^+^ cells in the DM and VL of P60 control and Satb2 CKO mice. *n* = 4 for each group. Statistical differences were determined by Student’s *t*-test, *p < 0.05, ***p < 0.001. Error bars represent S.E.M. 3V, third ventricle; ARC, arcuate nucleus; VMH, ventromedial hypothalamic nucleus. Scale bars = 100 μm in **a**, **a’**, 100 μm in **b**, **b’**, 100 μm in **c**–**e’**, 100 μm in **f**–**h’**.

Several hypothalamic nuclei including the ARC sense nutrient and endocrine cues and coordinate metabolic responses [21]. Our previous study showed that Satb2 is expressed in the ARC in adult mice [15]. Thus, the dysregulation of metabolism in Satb2 CKO mice prompted us to examine if there are any changes of the neuron types involved in metabolic activity, including POMC, NPY/AgRP, and tyrosine hydroxylase (TH, a marker for DA neurons) positive neurons. Using immunostaining, we first confirmed the expression of Satb2 in the ARC of control mice and verified the deletion of this gene in Satb2 CKO mice at P60 (Fig. 2a, a’). The Nissl-stained cellular architecture of the ARC was well maintained in Satb2 CKO mice relative to controls (Fig. 2b, b’). Then, we determined the neuron types by in situ hybridization (ISH). The quantitation results showed that the numbers of POMC^+^ (Fig. 2c, c’, i) and NPY^+^ (Fig. 2d’, d”, j) neurons were comparable in the ARC between control and Satb2 CKO mice, but the ISH signals of NPY with the same duration for signal development (1 h) was stronger in the ARC of Satb2 CKO mice than that in control (Fig. 2d, d”, k). Similar results were obtained for the ISH of AgRP in ARC (Supplementary Fig. S1). The number of TH^+^ neurons was significantly reduced in both DM and VL parts of ARC in Satb2 CKO mice, although the reduction in the VL was less severe than DM (Fig. 2e, e’, l). To exclude the possibility that the reduction is caused by the inability of TH expression itself rather than decreased neuron number, we examined the expression of other DA neuron markers including vesicular monoamine transporter 2 (VMAT2), aromatic l-amino acid decarboxylase (AADC), and dopamine transporter (DAT). Similar to the changes in TH expression, the expression of VMAT2, AADC and DAT were also dramatically decreased in the ARC of Satb2 CKO mice (Fig. 2f–h’). Thus, the number of DA neurons is indeed reduced with no obvious changes in the numbers of NPY^+^ and POMC^+^ neurons in the ARC of Satb2 CKO mice.

### Satb2 is expressed in adult and developing ARC DA neurons

**Fig. 3:**
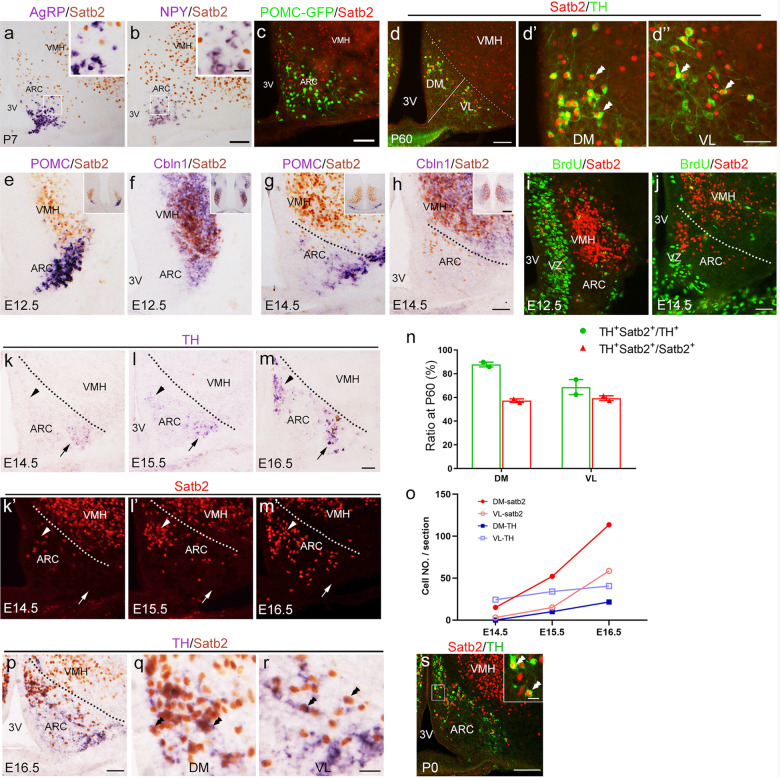
Satb2 is expressed in developing and adult ARC DA neurons. **a**, **b** Immunostaining of Satb2 combined with ISH of AgRP or NPY in wild-type mice. No colocalization of Satb2 with these two peptides is observed. **c** Satb2 is not detected in POMC^+^ neurons in the ARC of POMC-GFP transgenic mice. **d**–**d”** Double immunostaining of Satb2 and TH in the ARC of adult mice. **d’**, **d”** High magnification of the DM and VL in **d**. **e**–**h** Double staining of Satb2 (immunostaining, brown) with POMC or Cbln1 (ISH, dark blue) in the ventral hypothalamus at E12.5 (**e**, **f**) and E14.5 (**g**, **h**). At E12.5, all Satb2^+^ cells are located within the Cbln1^+^ presumptive VMH but not in POMC^+^ presumptive ARC (**e**, **f**). At E14.5, a few Satb2^+^ cells are located ventrally to the Cbln1^+^ VMH domain and within the POMC^+^ area (**g**, **h**). **i**, **j** Satb2^+^ cells are located outside the BrdU^+^ VZ in the ventral hypothalamus at E12.5 and E14.5. **k**–**m** Expression of TH mRNA in the ARC at E14.5, E15.5, and E16.5. TH mRNA is expressed in the VL (arrow) only at E14.5 (**k**), emerged in the DM (triangle) at E15.5 (**l**), and increased in both DM and VL at E16.5 (**m**). **k’**–**m’** Expression of Satb2 in the ARC at E14.5, E15.5, and E16.5. Satb2 is expressed in the DM (triangle) only at E14.5 (**k’**), emerged in the VL (arrow) at E15.5 (**l’**), and increased in both DM and VL at E16.5 (**m’**). **n** The percentages of double-labeling cells among the TH^+^ or Satb2^+^ cells in the ARC, *n* = 2. **o** Cell counts of Satb2^+^ and TH^+^ cells in DM and VL of ARC at E14.5, E15.5, and E16.5. **p**–**r** Double labeling of TH (dark blue) and Satb2 (brown) in the ARC at E16.5. **q**, **r** High magnification of the DM and VL in **p**, and TH/Satb2 double-labeled neurons are indicated with double triangles. **s** Double immunostaining of Satb2 and TH in the ARC and the insert shows Satb2/TH double-labeled neurons at P0. Error bars represent S.E.M. 3V third ventricle; ARC arcuate nucleus; DM dorsomedial part of ARC; VL ventrolateral part of ARC; VMH ventromedial hypothalamic nucleus; VZ ventricular zone. Scale bars = 100 μm in **a**–**c**, 25 μm in inserts of **a**, **b**, 100 μm in **d**, 50 μm in **d’**, **d”**, 50 μm in **e**–**h**, 200 μm in inserts of **e**–**h**, 50 μm in **i**, **j**, 50 μm in **k**–**m’**, 50 μm in **p**, **s**, 20 μm in **q**, **r**.

To explore why only TH expression was reduced in the ARC, we checked the colocalization of Satb2 with these genes in wild-type mice. Immunostaining of Satb2 combined with ISH of AgRP or NPY showed no colocalization of Satb2 with these peptides (Fig. 3a, b). Meanwhile, Satb2 was not detected in POMC^+^ neurons in the ARC of POMC-GFP mice [22] (Fig. 3c). Consistent with our previous data [15], TH/Satb2 double-labeled neurons were observed in the ARC at P60 (Fig. 3d–d”). We quantified the proportion of Satb2/TH-coexpressing neurons, and about 88% of TH^+^ neurons expressed Satb2, which corresponded to about 57% of Satb2^+^ neurons in the DM. In the VL, about 69% of TH^+^ neurons expressed Satb2, which was present in about 59% of Satb2^+^ cells (Fig. 3n). These data indicate that Satb2 is expressed in ARC DA neurons in both DM and VL with different proportions. Taken together, these data suggest an autonomous role of Satb2 in regulating the development of ARC DA neurons.

We next started to explore the expression pattern of Satb2 in developing ARC DA neurons. We investigated its expression in the ARC at different embryonic stages in combination with two specific markers Cbln1 [23] and POMC [24], which label the ventromedial hypothalamic nucleus (VMH) and ARC, respectively. Satb2 was first observed in the presumptive hypothalamus at E12.5 (Fig. 3e, f), and the vast majority of Satb2^+^ cells were located dorsally to POMC^+^ ARC domain (Fig. 3e) and within the Cbln1^+^ VMH domain (Fig. 3f). At E14.5, in addition to the presence of numerous Satb2^+^ cells in the VMH, a few Satb2^+^ cells were found to be located ventrally to the Cbln1^+^ domain and within the ARC region containing POMC^+^ cells (Fig. 3g, h). To determine Satb2 is expressed in progenitors or postmitotic cells, BrdU was injected intraperitoneally 1 h before the sacrifice of the pregnant mice. No Satb2^+^ cells were labeled with BrdU at E12.5, E14.5, and E16.5 (Fig. 3i, j, and data not shown), indicating that Satb2 is expressed in postmitotic ARC neurons.

We next investigated the expression of Satb2 in comparison with that of TH at different embryonic stages. Immunostaining of Satb2 was performed in adjacent sections of those processed for detection of TH mRNA at E14.5, 15.5, and 16.5. TH expression was initiated in the VL at E14.5 and DM at E15.5 in the ARC (Fig. 3k–m). However, Satb2 expression was first detected in the ARC at E14.5, and it was located in the DM only (Fig. 3k’, o). Satb2 was first detected in the VL at E15.5 and its expression was increased in both DM and VL at E16.5 (Fig. 3l’, m’, o). Colocalization of Satb2 and TH was observed at E16.5 and P0 (Fig. 3p–s). This unique expression pattern continued to P0 (Fig. 3s) and P60 (Fig. 3d). Thus, the initiation of Satb2 expression in the DM occurs earlier (i.e., E14.5) than that of TH (i.e., E15.5), but those in the VL show the opposite way during embryonic development.

### Satb2 is required for early differentiation of DA neurons in DM and for maintenance of DA neurons in VL

**Fig. 4:**
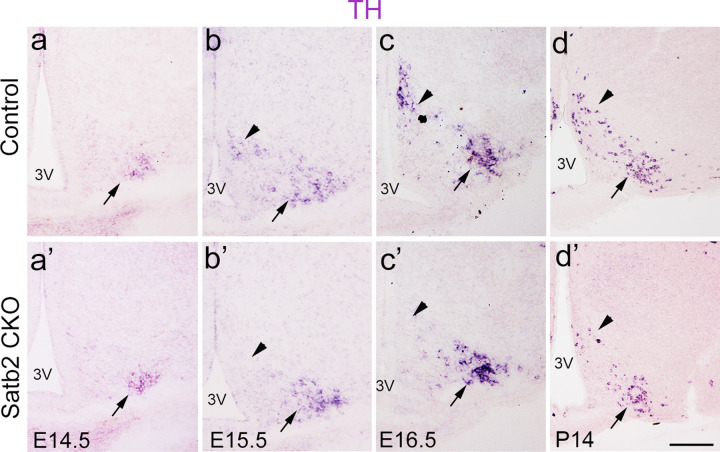
Satb2 is required for the differentiation of DA neurons in DM and for the maintenance of DA neurons in VL. **a**–**c’** Comparison of TH expression in the ARC of control and Satb2 CKO mice at different embryonic stages. TH mRNA is detected in the VL (arrows) in both control (**a**–**c**) and Satb2 CKO (**a’**–**c’**) embryos with no obvious differences at E14.5, E15.5, or E16.5. TH mRNA is present in the DM (triangles) of control mice at E15.5 (**b**) and E16.5 (**c**) whereas no TH transcripts are found in age-matched Satb2 CKO mice (**b’**, **c’**). **d**, **d’** Reduction of TH expression is first detected in the VL (arrow) of Satb2 CKO mice at P14, when few TH^+^ neurons are present in the DM (triangle) relative to controls. 3V third ventricle. Scare bars = 100 μm in **a**–**d’**.

The decline of DA neurons in adult Satb2 CKO mice might be due to defective generation and/or impaired maintenance. To figure out which was the case, we examined TH expression at different embryonic stages in Satb2 CKO mice. TH transcript was detected in the VL of control mice at E14.5 (Fig. 4a), and a similar expression pattern was observed in Satb2 CKO embryos (arrows, Fig. 4a, a’), showing that the initiation of TH expression in the VL is not affected. At E15.5, TH expression in the VL was similar between control and Satb2 CKO mice, but a significant reduction was observed in the DM of Satb2 CKO mice (Fig. 4b, b’), suggesting that the initiation of TH expression in the DM is impaired. At E16.5, an apparent increase of TH transcripts was observed in the DM of control mice but not in CKO mice, while the expression of TH mRNA seemed to be unchanged in the VL of Satb2 CKO mice relative to controls (Fig. 4c, c’). An obvious reduction of TH expression in the VL was found in Satb2 CKO mice at P14, and few TH-expressing cells were observed in the DM at this stage (Fig. 4d, d’). Thus, defective DA neuron differentiation occurs in the DM as early as E14.5, while a failure of DA neuron maintenance starts in the VL at postnatal stages in Satb2 CKO mice.

### GAD67^+^, Sst^+^, GHRH^+^, and Galanin^+^ neurons remain unchanged in ARC of Satb2 CKO mice

**Fig. 5:**
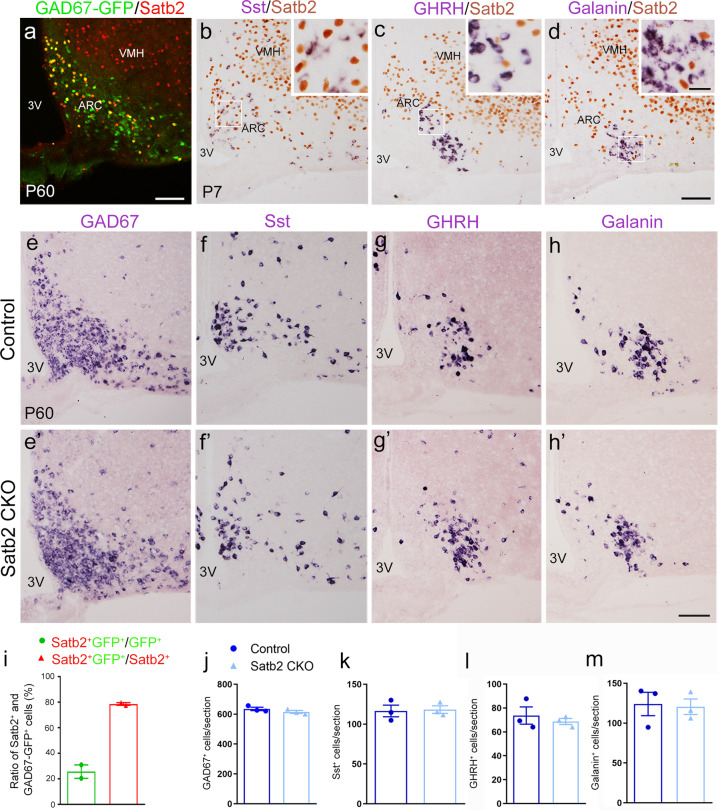
GAD67, Sst, GHRH, and Galanin-positive cells remain unchanged in ARC of Satb2 CKO mice. **a** Satb2 immunoreactivity is observed in a population of GFP^+^ cells in the ARC of adult GAD67-GFP transgenic mice. **b**–**d** Immunostaining of Satb2 combined with ISH of Sst, GHRH, and Galanin in wild-type mice. No colocalization of Satb2 with these peptides is observed. **e**–**h’** Expression GAD67, Sst, GHRH, and Galanin in the ARC is comparable between adult control (**e**–**h**) and Satb2 CKO mice (**e’**–**h’**). **i** The quantification of co-labeling of Satb2 and GAD67-GFP in the ARC of adult GAD67-GFP transgenic mice. *n* = 2. **j**–**m** The quantification of GAD67^+^ (**j**), Sst^+^ (**k**), GHRH^+^ (**l**), and Galanin^+^ (**m**) cells in the ARC shows no significant differences between the two groups. *n* = 3 for each group. Statistical differences were determined by Student’s *t*-test. Error bars represent S.E.M. 3V third ventricle; ARC arcuate nucleus; VMH ventromedial hypothalamic nucleus. Scale bars = 100 μm in **a**, 100 μm in **b**–**d**, 25 μm in inserts of **b**–**d**, and 100 μm in **e**–**h’**.

We have shown that the development of DA neurons is affected in Satb2 CKO mice with unchanged NPY^+^ and POMC^+^ populations. However, there are several other types of neurons in the ARC characterized by distinct neurotransmitters, neuroendocrine functions, or expression of specific neuropeptides, including GAD67, Sst, GHRH, and Galanin [25, 26]. We next examined if these neuronal populations were altered by double immunostaining or ISH combined with immunostaining in wild-type mice. The majority of Satb2^+^ neurons expressed GFP in the ARC of GAD67-GFP mice [27] (Fig. 5a, i). Similar to the cases of AgRP/NPY/POMC, there were no Satb2^+^ neurons expressing Sst, GHRH, or Galanin (Fig. 5b–d). The number of GAD67^+^ cells in the ARC of Satb2 CKO mice was not different from that of control mice (Fig. 5e, e’, j). As expected, cell counts showed that the numbers of Sst^+^, GHRH^+^, and Galanin^+^ neurons were not significantly changed in adult Satb2 CKO mice compared with controls (Fig. 5f–h’, k–m). Taken together, our data indicate that Satb2 is selectively involved in the development of ARC DA neurons.

As mentioned above, Satb2 is also expressed in the VMH (Fig. 3). However, the cellular architecture of VMH shown by Nissl staining was not apparently altered in adult Satb2 CKO mice (Fig. 2b, b’). Furthermore, several genes are specifically expressed in the VMH such as Cbln1, Nr5a1, and Sox14 [23, 28], and their expression was also comparable between control and Satb2 CKO mice (Supplementary Fig. S2), suggesting that the overall morphology of the VMH might not be significantly affected. In addition, cleaved Caspase3 immunostaining at E14.5, E16.5, and P0 did not detect significant differences of the positive cells in the ARC between control and Satb2 CKO mice (Supplementary Fig. S3).

### Serum prolactin level is increased in female Satb2 CKO mice

**Fig. 6:**
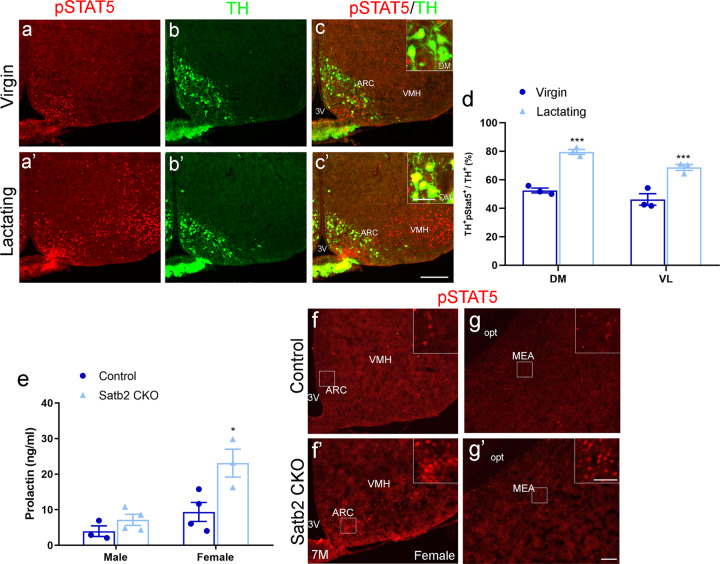
Serum prolactin level is increased in female Satb2 CKO mice. **a**–**c’** Double immunostaining of pSTAT5 and TH in the hypothalamus of virgin and lactating females. Expression of pSTAT5 is massively elevated in lactating dams comparing to virgin females. Inserts show the colocalization of TH and pSTAT5. **d** The colocalization of TH and pSTAT5 is significantly increased in DM and VL of ARC in lactating dams comparing to virgin females. *n* = 3 for each group. **e** The level of serum prolactin is increased in female Satb2 CKO mice relative to control, although the *p*-value does not reach a significant difference in the male group. For males, *n* = 3 for control and *n* = 4 for Satb2 CKO mice; for females, *n* = 4 for control and *n* = 3 for Satb2 CKO mice. **f**–**g’** A large number of pSTAT5^+^ cells are present in the ARC, VMH, and MEA of Satb2 CKO mice of seven months old (**f’**, **g’**), where they were hardly detected in age-matched control mice (**f**, **g**). Inserts show the enlarged pSTAT5^+^ cells. Statistical differences were determined by Student’s *t*-test, *p < 0.05, ***p < 0.001. Error bars represent S.E.M. 3V third ventricle; ARC arcuate nucleus; DM dorsomedial part of ARC; MEA medial nucleus of the amygdala; VMH ventromedial hypothalamic nucleus. Scale bar = 250 μm in **a**–**c’**, 20 μm in inserts of **c**, **c’**, 100 μm in **f**–**g’** and 50 μm in inserts of **f**–**g’**.

ARC DA neurons serve as an inhibitor in regulating the secretion of prolactin [29]. We thus set out to examine the expression of pSTAT5, a signal transducer and activator for prolactin secretion [30] in virgin and lactating females. Immunostaining showed that the expression of pSTAT5 was dramatically increased in the ARC and VMH in lactating dams compared with virgin females (Fig. 6a, a’), consistent with the notion that suckling stimulates the secretion of prolactin in lactation [29]. The proportion of pSTAT5 expression in DA neurons was also significantly increased in the DM and VL of ARC in lactating mice (inserts in Fig. 6c, c', d). Consistent with the inhibitory role of ARC DA neurons in regulating the secretion of prolactin, we found that the levels of serum prolactin were increased in adult female Satb2 CKO mice, although those in male CKO mice showed an increasing trend (Fig. 6e). Besides, pSTAT5 was abundantly expressed in the ARC, VMH and medial nucleus of the amygdala (MEA) of female Satb2 CKO mice (inserts in Fig. 6f’, g’), whereas few pSTAT5^+^ cells were scattered in these regions in control mice (inserts in Fig. 6f, g), which is consistent with the increase of prolactin secretion in Satb2 CKO mice. In addition, the size of the pituitary gland was reduced in Satb2 CKO mice compared with control mice (Supplementary Fig. S4a–c), which is consistent with the reduction of body weight in Satb2 CKO mice (Fig. 1a). The secretion of prolactin is regulated by ARC-dopamine through the D2 receptor (DRD2), which is expressed in both the intermediate lobe (IL) and anterior lobe (AL) of the pituitary gland [31]. ISH showed that the expression of Drd2 in the AL was slightly increased in Satb2 CKO mice (Supplementary Fig. S4d, e). Growth hormone (GH) is also generated and secreted in the AL of the pituitary gland [31], but the intensity of GH immunofluorescence was not obviously changed in the AL of Satb2 CKO mice compared with control, suggesting that the generation of GH may not be affected (Supplementary Fig. S4f, g). Collectively, it is very likely that reduced ARC DA neurons lead to elevated prolactin levels in Satb2 CKO mice.

### Dlx1 acts downstream of Satb2 in development of ARC DA neurons

**Fig. 7:**
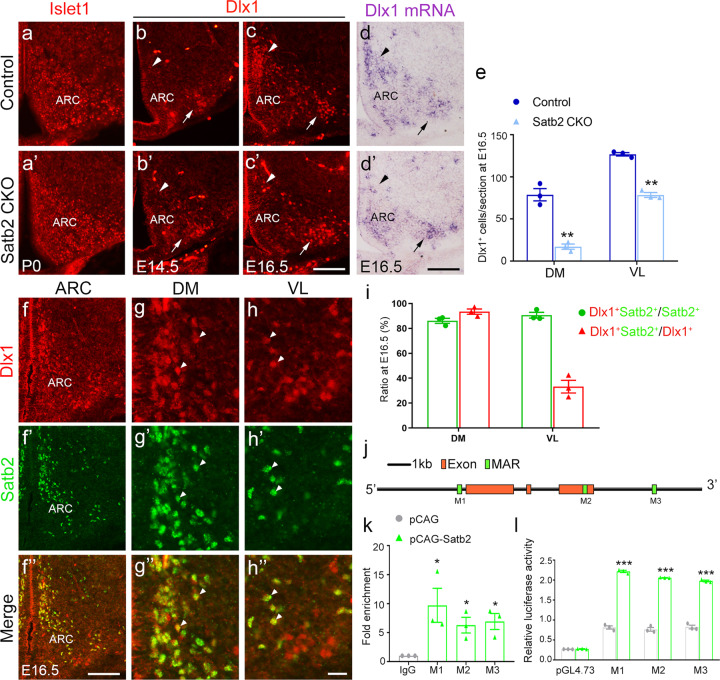
Dlx1 acts downstream of Satb2 in ARC DA neurons. **a**, **a’** Expression of Islet1 is not significantly changed in Satb2 CKO mice relative to controls at P0. **b**, **b’** At E14.5, Dlx1 is only detected in the VL (arrows) but not DM (triangles) of ARC in both control mice and Satb2 CKO mice with no obvious differences. **c**, **c’** At E16.5, Dlx1 immunoreactivity is present in both DM (triangles) and VL (arrows) of ARC in control mice but dramatically reduced in the ARC particularly in its DM of Satb2 CKO mice. **d**, **d’** Dlx1 mRNA shows similar changes in the ARC of Satb2 CKO mice relative to controls. **e** Comparison of Dlx1^+^ cells in the DM and VL between control and Satb2 CKO mice at E16.5. *n* = 3 for each group. **f**–**h”** Double immunostaining of Dlx1 and Satb2 in the ARC of wild-type mice at E16.5 (**f**–**f”**), and Dlx1/Satb2 double-labeled neurons in the DM (**g**–**g”**) and VL (**h**–**h”**) are shown in high magnification. **i** The quantification of Dlx1/Satb2 double-labeled neurons in the DM and VL in E16.5 wild-type mice. *n* = 3. **j** Diagram of Satb2-binding MARs in Dlx1 genomic locus. **k** ChIP assay shows a remarkable enrichment of Satb2-binding MARs. *n* = 3 animals. **l** Luciferase reporter assay shows that the transcriptional activities of three Dlx1 MARs are significantly increased in the presence of Satb2. *n* = 3 for each group. Statistical differences were determined by Student’s *t*-test, *p < 0.05, **p < 0.01. Error bars represent S.E.M. ARC arcuate nucleus; DM dorsomedial part of ARC; MAR matrix attachment region; VL ventrolateral part of ARC. Scale bars = 100 μm in **a**–**c’**, 100 μm in **d**, **d’**, 100 μm in **f**–**f”**, and 20 μm in **g**–**h”**.

Previous studies have identified several transcription factors that are involved in the development of the ARC [7–10, 32]. We found that Nkx2.1 expression in the ventral hypothalamus was not altered in the CKO mice relative to controls (Supplementary Fig. S5a, a’). In addition, the expression of Islet1 was not changed either in the ARC of Satb2 CKO mice (Fig. 7a, a’). Further, Mash1 expression was not changed in Satb2 CKO mice (Supplementary Fig. S5b, b’). We next investigated the expression of Dlx1 in the ARC. Dlx1 expression was not different in the ARC between control and Satb2 CKO mice at E14.5 (Fig. 7b, b’). However, its expression was dramatically reduced in the DM with a less severe reduction in the VL of Satb2 CKO mice at E16.5 (Fig. 7c, c’), which resembled the alternation of TH expression in the ARC. This change was confirmed by ISH for Dlx1 mRNA (Fig. 7d, d’). The quantitation of Dlx1^+^ cells in the DM and VL of ARC at E16.5 is shown in Fig. 7e. The loss of DA neurons in the ARC has been reported in Dlx1 KO and CKO mice [10, 33], and here we showed that Dlx1 expression depends on Satb2 in the ARC, raising the possibility that Satb2 regulates ARC DA neuron development by Dlx1.

To explore this, we examined the colocalization of Dlx1 and Satb2 in the ARC. The majority of Dlx1^+^ cells expressed Satb2 in the DM and to less extent in the VL at E16.5 (Fig. 7f–h”). In the DM, 93% of Dlx1^+^ cells expressed Satb2, while 86% of Satb2^+^ cells expressed Dlx1. However, in the VL, 33% of Dlx1^+^ cells expressed Satb2, while 90% of Satb2^+^ cells expressed Dlx1 (Fig. 7i). In addition, we also found that most TH^+^ cells expressed Dlx1 in both DM and VL of ARC at P7 (Supplementary Fig. S6a–c”). Satb2 is known to interact with nuclear matrix attachment regions (MAR) and regulate transcription of downstream genes via direct binding to MAR sites [34]. To determine whether Satb2 protein directly binds to MAR sites of the Dlx1 gene and regulates its expression, we performed ChIP and dual-luciferase reporter assays. According to theoretical prediction (http://genomecluster.secs.oakland.edu/marwiz/), there are three putative MAR sites in the Dlx1 genome (Fig. 7j). ChIP assay showed significant enrichment of these Dlx1-MAR sequences after Satb2 antibody incubation (Fig. 7k), indicating a direct association of Satb2 protein with Dlx1-MAR sequences. Then the dual-luciferase reporter assay was performed to examine the transcription activity of Satb2 in regulating Dlx1 expression. When Dlx1-MAR sequences were cloned into pGL4.73 plasmid, luciferase activity was increased when Satb2 cDNA was co-transfected (Fig. 7l). Taken together, these data suggest that Dlx1 may function as a downstream gene of Satb2 in regulating the development of DA neurons in the ARC.

## Discussion

The ARC has a critical role in the regulation of feeding and energy balance. Two distinct and functionally antagonistic types of neurons positive for POMC or AgRP/NPY are ‘first-order’ neurons in the ARC, which receive and integrate metabolic signals [35]. We did not detect any changes in the cell number of POMC or AgRP/NPY neurons in Satb2 CKO mice (Fig. 2). Recently, DA neurons that control the activity of adjacent POMC and AgRP/NPY neurons and thus are involved in the regulation of energy homeostasis have been identified in the ARC [6]. Consistently, we found that DA neurons are significantly decreased in Satb2 CKO mice (Figs. 2 and 4). Besides, the ARC DA neurons are well-known for a suppressor role in the regulation of the secretion of prolactin via the tuberoinfundibular pathway [29]. We found that the serum prolactin level is increased, and prolactin-responsive neurons shown by pSATA5 are also increased in the brain of Satb2 CKO mice, similar to the elevation of pSTAT5 in lactating females (Fig. 6). Although the expression of GH is unchanged (Supplementary Fig. S4), the reduced size of the pituitary gland may reflect defective synthesis and secretion of other hormones in the gland. Thus, further studies are needed to explore if the other hormones are changed and their contributions to dysregulation of body weight and metabolism observed in Satb2 CKO mice.

Previous studies have identified several proneural genes that are involved in controlling the development of ARC in the hypothalamus, such as Mash1 [7], Ngn3 [36], and Nkx2.1 [8]. Our results showed that Satb2 is only expressed in postmitotic neurons of ARC (Fig. 3), and expression of Nkx2.1 and Mash1 is not significantly changed in Satb2 CKO mice (Supplementary Fig. S5). Given that the apoptosis is comparable in the ARC between control and Satb2 CKO mice, it thus is likely that the reduction of DA neurons in the ARC is caused by defective differentiation of postmitotic DA neurons in the absence of Satb2. However, it should also be noted that the DA neurons in the ARC are not equally affected in Satb2 CKO mice, as shown by a more drastic reduction of DA neurons in the DM than that in the VL. A higher proportion of DA neurons in the DM expresses Satb2 in comparison with that in the VL (88% in DM versus 57% in VL), and this may account for the discrepancy, indicating that Satb2 is required for the differentiation of DA neurons in a cell-autonomous way. On the other hand, Satb2 seems to regulate the differentiation of ARC DA neurons in different ways. The initiation of Satb2 is prior to and required for that of TH in the DM. In the VL, however, Satb2 and TH initiate in an opposite way, and the initiation of TH is normal in Satb2 CKO mice. The reduction of DA neurons in the VL occurs during early postnatal days and this supports the idea that Satb2 is required for the maintenance of the neurotransmitter phenotype of DA neurons in the VL. Nevertheless, Satb2 plays a pivotal and specific role in the development of DA neurons in the ARC.

In summary, we found that the body weight and metabolic rate are altered in Satb2 CKO mice, possibly due to the reduction of ARC DA neurons. We also showed that Satb2 regulates the development of ARC DA neurons through potential downstream gene Dlx1. We finally revealed one of the functional consequences of impaired ARC DA neurons shown by the increased level of serum prolactin.

## Materials and methods

### Animals

All mice were housed in a specific pathogen-free animal facility under a normal 12 h light, 12 h dark cycle with ad libitum access to normal chow and water. To conditionally knock out Satb2 gene in the brain, Nestin-Cre mice [20] were crossed with floxed Satb2 mice [19] to delete exon 4 (Nestin-Cre;Satb2^flox/flox^, referred to as Satb2 CKO thereafter). POMC-GFP and GAD67-GFP mice were used as described previously [22, 37]. For virgin and lactating mice, sexually naïve females were used. The lactating group was mated with males and the virgin group was not. About 10 days after the birth of pups, the lactating mice were euthanized for experiments, along with the virgin mice.

### Metabolic expenditure measurements

The control and Satb2 CKO mice of 10-month-old were placed in a Comprehensive Laboratory Animal Monitoring System (CLAMS, Columbus Instruments, Columbus, OH) for the evaluation of metabolism. Oxygen consumption (VO2) and carbon dioxide expiration (VCO2) were measured. The respiratory exchange ratio (RER) and energy expenditure were calculated based on the oxygen and carbon dioxide data.

### Immunohistochemistry, BrdU labeling, and in situ hybridization

The stage of mouse embryos was determined by taking the morning when the copulation plug was observed as embryonic day 0.5 (E0.5). Anesthetized embryos and mice were perfused transcardially with phosphate-buffer saline (PBS; pH7.4) first then with 4% paraformaldehyde (PFA). All brains were fixed in 4% PFA overnight and cryoprotected in PBS containing 30% sucrose. Brains were cut into 20-μm-thick sections using a cryostat (CM1950; Leica, Wetzlar, Germany).

For immunohistochemistry, brain sections were then incubated with rabbit anti-Satb2 (1:300; ab92446, Abcam, Cambridge, UK), mouse anti-Satb2 (1:200; ab51502, Abcam), rat anti-BrdU (1:300; OBT0030, Accurate Chemical & Scientific Corporation, Carle Place, NY), Cleaved Caspase3 (1:300; #9661, Cell Signaling Technology, Danvers, MA), pSTAT5 (1:500; #9314, Cell Signaling Technology), Islet1 (1:100; 40.2D6, DSHB, Iowa City, IA), mouse anti-TH (1:10,000; T2928, Sigma, Burlington, MA), rabbit anti-Dlx1 (1:500; GTX108804, GeneTex, Irvine, CA) or rabbit anti-GH (1:100; AFP5672099, NIDDK-NHPP, La Jolla, CA) antibodies at 4 °C overnight, then incubated with biotinylated or fluorophore-conjugated secondary antibodies (1:500; biotinylated horse anti-rabbit or biotinylated horse anti-goat from Vector Laboratories, Burlingame, CA; Alexa Fluor 488 donkey anti-rat or Alexa Fluor 488 horse anti-mouse from Jackson ImmunoResearch, West Grove, PA) for 3 h, followed by incubation with streptavidin-Cy3 (1:1000; Jackson ImmunoResearch) and Hoechst 33258 (1:2000; Sigma) for 1 h.

For the BrdU assay, pregnant mice were received one pulse of BrdU (100 mg/kg; Sigma) by intraperitoneal injection and killed 1 h later. Brain sections were sequentially subjected to citrate buffer (0.01 M, pH6.0) at 95 °C for 10 min, HCl (2 N) at 37 °C for 30 min, and sodium borate (0.1 M) at room temperature for 10 min. Treated sections were immunostained with rat anti-BrdU antibody (1:300; Accurate Chemical & Scientific Corporation) as described above.

In situ hybridization (ISH) was performed as described previously [38]. The following RNA probes were used: POMC, Cbln1, TH, VMAT2, AADC, DAT, Sst, NPY, AgRP, GHRH, Galanin, GAD67, Nr5a1, Sox14, Dlx1, Mash1, Nkx2.1, and Drd2.

For double labeling of ISH and Satb2 immunostaining, sections were performed with ISH procedures first. After visualization for mRNA, sections were incubated with a rabbit anti-Satb2 antibody (1:300; ab92446, Abcam) at 4 °C overnight, followed by a biotinylated horse anti-rabbit antibody (1:500; Vector Laboratories) for 2 h. Sections were then processed using the Vectastain Elite ABC kit (Vector Laboratories) for 1 h and immunoreactivity was visualized by incubation with diaminobenzidine and H_2_O_2_.

### Nissl and H&E staining

For Nissl staining, brain sections were placed in xylene for 30 m, followed by 100% ethanol, 95% ethanol, and 80% ethanol, for 5 m each. The sections were incubated in 1% crystal violet for 1 h, then sequentially incubated in 80% ethanol, 95% ethanol, 100% ethanol and 100% ethanol for 5 m each, followed by twice rinses in xylene for 5 m each. Finally, the sections were mounted in neutral balsam. Pituitary sections were rehydrated and subjected to H&E staining as previously described [39].

### Enzyme-linked immunoassay analyses

Enzyme-linked immunoassay analyses were performed to quantify levels of prolactin in the serum. Blood was collected from the adult mouse heart and placed at 4 °C for 30 min, followed by centrifuging at 2000 × *g* for 20 min at 4 °C. The top layer (serum) of the sample was collected and prolactin concentration was measured using an ELISA kit (ab214572, Abcam), according to the manufacturer’s instructions.

### Chromatin immunoprecipitation (ChIP)

ChIP assay was performed using hypothalamic tissues of wild-type mice (E16.5) according to the manufacturer’s protocol of SimpleChIP Plus Sonication Chromatin IP Kit (#56383, Cell signaling technology). The supernatant was immunoprecipitated with a rabbit anti-Satb2 antibody (ab34735, Abcam). Co-precipitated DNA was purified and relative DNA abundance of regions of interest was measured by real-time PCR. The following primers were used for PCR: Dlx1-MAR1: 5′AGCCTCACCTTGTGGTTTGG3′ and 5′GCAGCATGTTGGTTGGAGC3′; Dlx1-MAR2: 5′CCAGGTCGCTTTAAAGTAAGACAC3′ and 5′CAACAGAACGGAAAGAGGCTAAGC3′; Dlx1-MAR3: 5′GTGACTTCTCTAGCAAGGAGAC3′ and 5′CTAAAGACCGCCTTCCTTGAGC3′. Three animals were used for the ChIP assay for each group.

### Luciferase reporter assay

For the luciferase reporter assay, a pGL4 dual transcription activity luciferase system (Promega, Madison, WI) was used. Medulloblastoma cell line DAOY was cultured in 24-well plates and transfected by Lipofectamine 2000 (Invitrogen, Waltham, MA). DAOY cells for each well were transfected with 400 ng Dlx1-MAR(1–3)-Luc reporter constructs, 50 ng pGL4.73 construct (as an internal control), together with 400 ng pCAG-Satb2 or its empty constructs. The cells were harvested for the luciferase reporter assays 30 h after transfection. Three cultures for each group were used.

### Statistics

The ARC area adjacent to the third ventricle on both sides was chosen for cell count. The numbers of neurons indicated with TH, Satb2, GFP, NPY, Galanin, POMC, GAD67, and Dlx1 were counted in control and Satb2 CKO mice (*n* = 3 for each group). The average optical density (OD) of the NPY ISH signal was measured by Image Pro-Plus 6.0 (*n* = 3 for each group). Origin8 software was used for statistical analysis. All data were tested for normal distribution and homogeneity of variance. Comparisons were performed using the two-tailed Student’s *t*-tests. Statistical significance is displayed as *p < 0.05, **p < 0.01, ***p < 0.001, ****p < 0.0001.

## Supplementary information

Supplemental Material

## Data Availability

All data generated in this study are included either in this article or in the Supplementary Information files.
