# Supplementary material for: Satb2 regulates the development of dopaminergic neurons in the arcuate nucleus by Dlx1
Source: Cell Death Dis. 2021 Sep 25;12(10):879. doi: 10.1038/s41419-021-04175-9 (PMC8464595; doi:10.1038/s41419-021-04175-9)
Supplement: Supplementary file 1 — Supplemental Material [file 41419_2021_4175_MOESM1_ESM.docx]

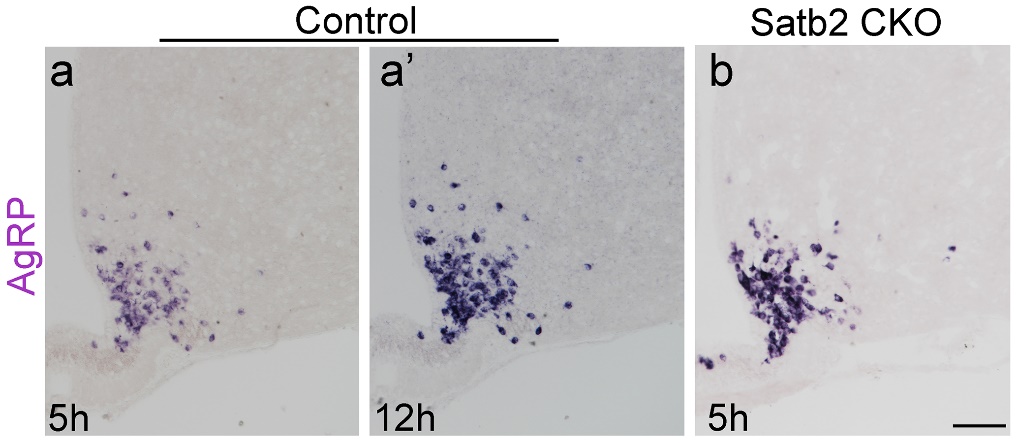


**Figure S1.** **AgRP^+^ neurons are not affected in the ARC of Satb2 CKO mice.** (a-b) ISH shows that the number of AgRP^+^ neurons is comparable between control and Satb2 CKO mice (a’, b), but the intensity of *in situ* hybridization signals is higher in the ARC of Satb2 CKO mice than that in the control at the same duration of signal development (a, b). Note that (a, a’) are the same section with different duration of ISH signal development. Scale bar = 100 μm in (a-b).


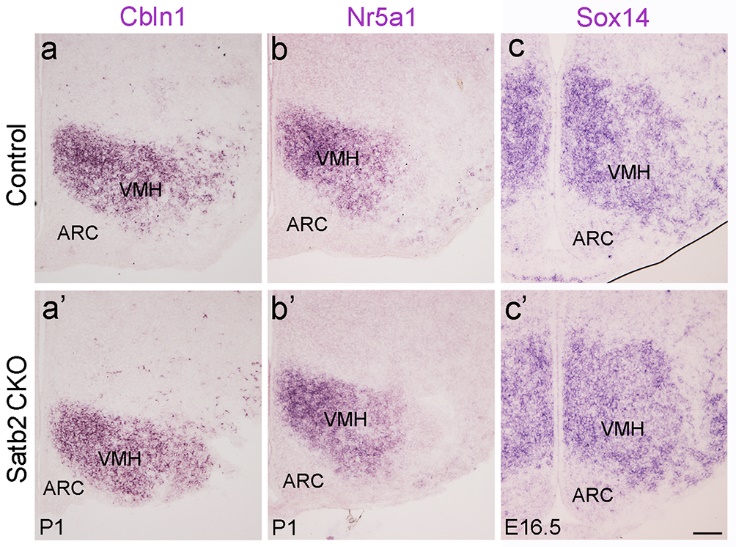


**Figure S2. VMH genes are not affected in Satb2 CKO mice.** (a-c’) The expression of Cbln1, Nr5a1, and Sox14 is comparable in the ventromedial hypothalamic nucleus (VMH) between control and Satb2 CKO mice. ARC, arcuate nucleus. Scale bar = 100 μm in (a-c’).


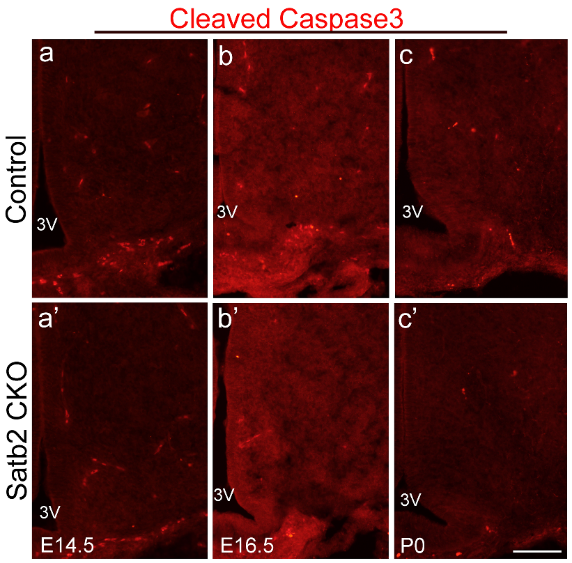


**Figure S3. Cell apoptosis is comparable between control and Satb2 CKO mice at E14.5, E16.5 and P0.** (a-c’) Immunostaining of cleaved Caspase3 shows the apoptotic cells are comparable between control and Satb2 CKO mice at E14.5, E16.5 and P0. Scale bar = 100 μm in (a-c’).


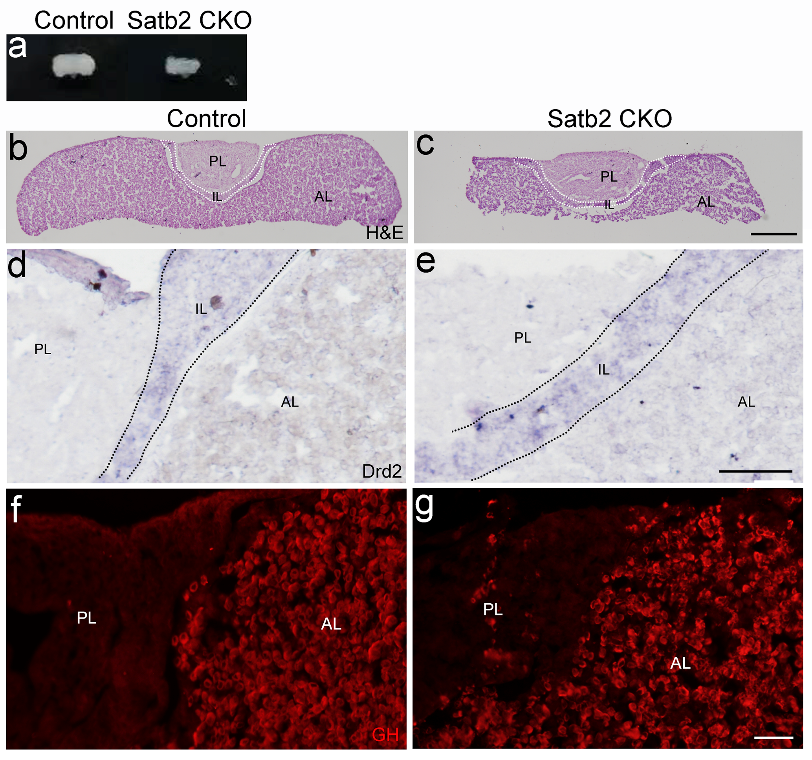


**Figure S4.** **Smaller pituitary gland with slightly increased** **Drd2 and unchanged GH expression in Satb2 CKO compared with control mice.** (a-c) A general view (a) and section (b, c, H&E staining) of adult pituitaries from control and Satb2 CKO mice. Satb2 CKO mice showed a reduced pituitary compared with control mice. (d, e) Expression of Drd2 in the anterior lobe (AL) revealed by ISH is slightly increased in Satb2 CKO mice. (f, g) Immunostaining of GH in the pituitaries of control and Satb2 CKO mice. No apparent change was observed in the expression of GH in AL at the cellular level in Satb2 CKO mice. Drd2, dopamine receptor D2; GH, growth hormone; IL, intermediate lobe of pituitary; PL, posterior lobe of pituitary. Scale bars = 500 μm in (b, c), 100 μm in (d, e), 50 μm in (f, g).


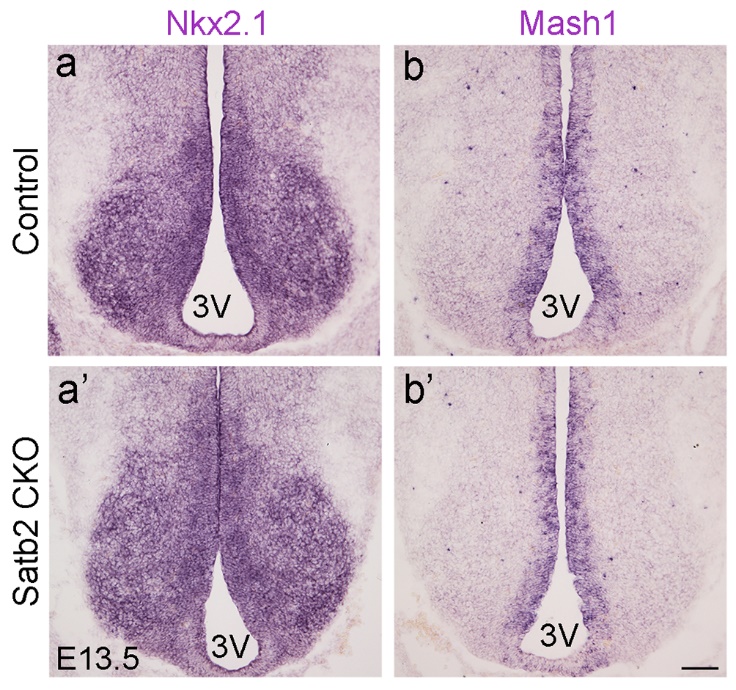


**Figure S5. Expression of Nkx2.1 and Mash1 in VZ is not affected in Satb2 CKO mice.** (a-b’) The expression of Mash1 and Nkx2.1 is comparable between control and Satb2 CKO mice at E13.5. 3V, third ventricle. Scale bar =100 μm in (a-b’).


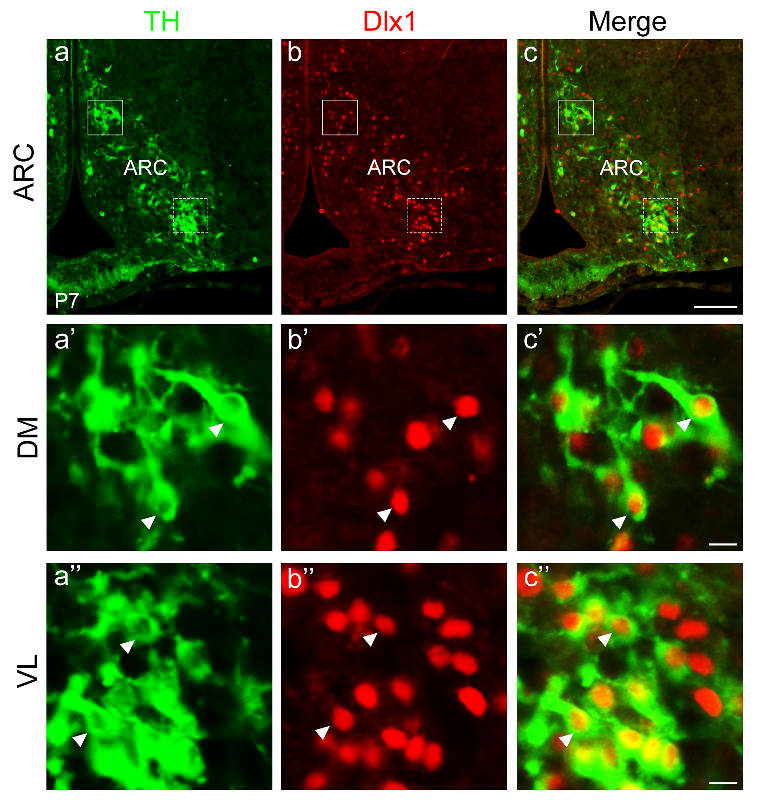


**Figure S6. TH and Dlx1 are colocalized in both DM and VL of ARC.** (a-c’’) Double immunostaining of TH and Dlx1 in the ARC of wild-type mice at P7. TH/Dlx1 double-labeled neurons in the DM (a’-c’) and VL (a’’-c’’) are shown in high magnification of solid- and dashed-box in (a-c), respectively. Scale bars = 100 μm in (a-c), 10 μm in (a’-c’), 10 μm in (a’’-c’’).
